# The Common Task

**Published:** 1908-01-11

**Authors:** 


### THE COMMON TASK.

**Correspondence and Queries** for this section should be sent to the Editor of The Hospital, 28 Southampton Street, Strand, London, and marked "Nursing Administration."

### Total Abstinence for Nurses.

At the successful meetings of the Women's Total Abstinence Union recently held the nurses' branch of this intensely zealous Society was well represented. Perhaps the best inducement for nurses to join the Union lies in the consideration that to be a total abstainer is an excellent factor in the nurse, because it enables her to speak with experience on the want of any necessary connection between good health and alcohol. Many women, and these not always without education, believe that they ought to take as much alcohol as they can get in the child-bearing period, or when any strain falls upon them; and the object-lesson of a nurse in perfect health, doing harder work than they are called upon to do themselves, and yet abstaining from alcohol, is worth more than hours of talk as a help to temperance. Quite independently of the example set, the abstainer profits undeniably in digestion and comfort. Whatever alcohol may do for people of leisure, it is ill-adapted as a beverage under almost any form, except that of exceedingly light wine, for the use of those who have to be stirring shortly after meals. We cordially wish the Nurses' Total Abstinence Society success in the campaign they are waging against ignorant self-indulgence.

### The Health of Night Nurses.

It is much a question of personal idiosyncracy whether a nurse experiences any deterioration of health during her term of night service. Some nurses greatly prefer night work, and find in some curious way that it leaves them with more spare time than ordinary day work. And of these are made the best night superintendents, who, since their term of office commonly lasts a considerable time, can only perform their duties satisfactorily if their vitality is not lowered by the enforced change of habits demanded by the post. But instances are not uncommon of women who can never sleep restfully at an unaccustomed hour and who languish through the time perforce spent on night duty in discomfort so acute as to threaten in the long run a nervous breakdown. Experiences of this kind have induced certain well-meaning guardians to introduce a system of short shifts, the nurses taking night-duty turn and turn about. Truth to tell, there could hardly be devised a worse expedient. There are many people who are so emancipated from habit as not to find the first occasions of day-time sleep wakeful and unrestful. By mixing day and night work the body never gets habituated to the new conditions, and the repose which is obtained is the product of exhaustion rather than a natural disposition visiting unconsciously the tired eyes. The remedy for the night nurse who cannot sleep is complete isolation from noise and light with more attention to food. It is indigestion which is, after all, the night nurse's great enemy, too much tea drinking, and heavy food partaken at unaccustomed hours.

THE HOSPITAL January 11, 1908.

